# The Basics of Penicillin Allergy: What A Clinician Should Know

**DOI:** 10.3390/pharmacy7030094

**Published:** 2019-07-17

**Authors:** Louis Lteif, Lea S. Eiland

**Affiliations:** 1 System Pharmacy Services, Sharp HealthCare, 8695 Spectrum Center Blvd, San Diego, CA 92123, USA; 2 Department of Pharmacy Practice, Harrison School of Pharmacy, Auburn University, 1321 Walker Building, Auburn, AL 36849, USA

**Keywords:** allergy, beta-lactam, hypersensitivity, penicillin

## Abstract

Antimicrobials in the penicillin class are first line treatments for several infectious diseases in the pediatric and adult population today. In the United States, patients commonly report having a penicillin allergy, with penicillin being the most frequent beta-lactam allergy. However, very few patients experience a clinically significant immune-mediated allergic reaction to penicillin. If a true penicillin allergy exists, cross-reactivity to other beta-lactam antimicrobials may occur. Mislabeling patients with penicillin allergy can lead to a higher utilization of second line antimicrobial agents, potentially increasing costs and resistance due to a larger spectrum of activity. Pharmacists play an essential role in inquiring about patient specific reactions to presumed medication allergies and developing a further assessment plan, if needed, to determine if the medication allergy is real.

## 1. Introduction and Incidence of Penicillin Allergy

Patient assessment of drug allergies is a standard practice of pharmacists. The most commonly reported drug class allergy is to penicillins, with penicillin being the most common beta-lactam allergy [1,2]. Approximately 8–10% of people in the United States report a penicillin allergy [2,3]. Hypersensitivity reactions are common with penicillins [4]. However, very few patients have a clinically significant immune-mediated allergic reaction. It is imperative for pharmacists to ask about patient specific reactions to presumed medication allergies when gathering a patient history. This information is essential to determine if the medication allergy is real or if further assessment is needed. Less than 5% of patients who claim a penicillin allergy will likely experience a reaction if provided an oral challenge using a therapeutic dose [5] and approximately 95% of patients with a reported penicillin allergy will have a negative penicillin skin test [6]. In addition, IgE antibodies wane over time, resulting in negative skin tests in most patients after 10 years [7]. These patients are at low risk of becoming resensitized if exposed to penicillin in the future [8,9].

## 2. Indications

Penicillin was introduced in the 1940s [10]. Several drugs have evolved from penicillin and all are classified by their antimicrobial activity. In general, penicillins are active against gram-positive cocci, with some drugs having additional gram-negative coverage and even extended spectrum gram-negative coverage. Despite the rise of resistant organisms, penicillin and others within the class continue to remain a key antimicrobial treatment for several infections in the pediatric and adult population.

Natural penicillins, such as penicillin G potassium and penicillin V potassium, remain the drugs of choice for infections such as syphilis [11], penicillin-sensitive streptococcal and enterococci endocarditis [12], and group A beta-hemolytic streptococcus pharyngitis [13]. Penicillinase-resistant penicillins (also called antistaphylococcal penicillins) are used primarily for skin and soft tissue infections [14]. Oxacillin, nafcillin, and dicloxacillin are drugs of choice for diverse infections caused by methicillin-susceptible *Staphylococcus aureus*. Aminopenicillins have additional activity against gram-negative organisms such as *Escherichia coli* and *Haemophilus influenzae*; thus, may be used first line for gram-positive or gram-negative infections such as acute otitis media [15] and rhinosinusitis [16]. Ampicillin is the drug of choice for *Listeria monocytogenes* [17] and sensitive Enterococcus species [18]. Amoxicillin is the recommend first line agent for prophylaxis of bacterial endocarditis [18]. Amoxicillin-clavulanate’s spectrum includes anaerobic organisms making it the drug of choice for human and animal bites [14]. Antipseudomonal penicillins such as piperacillin and ticarcillin have additional coverage of *Pseudomonas aeruginosa* and drug-resistant gram-negative rod organisms. They are commonly combined with a beta-lactamase inhibitor and prescribed for mixed infections or as empiric therapy of nosocomial infections.

## 3. Risk Factors

Outcomes analyzing potential host risk factors for penicillin allergies have been conducted. Intrapartum use of penicillin, amoxicillin, or ampicillin did not increase the risk of a child developing a penicillin allergy [19]. A small, prospective study determined age, sex, family history, and atopy were similar between patients with confirmed penicillin and amoxicillin allergy and patients without an allergy diagnosis [20]. Age, sex, and atopy also were not determined to be risk factors of a beta-lactam allergy in a study of 1865 children [21]. However, a large, retrospective study found an increased prevalence of a penicillin allergy in female versus male patients (OR = 1.82, *p* < 0.0001) [22]. Park and colleagues also found females were more likely than males to have a positive penicillin skin test reaction before (OR = 3.6, *p* < 0.001) and after adjusting for confounders (OR = 3.2, *p* = 0.001) [23]. Lastly, a small, adult study determined that a history of a penicillin allergy, an adverse drug reaction history to other medications (non-beta-lactam), and atopy were risk factors for a penicillin allergy [24]. The authors proposed a genetic influence on penicillin allergy. Larger, well-designed studies are needed to continue to assess patient risk factors for a penicillin allergy.

In addition, the development and magnitude of a penicillin allergic reaction may not correspond to the dose. A patient can experience a severe, anaphylactic reaction after administration of a low, or any, dose of the antibiotic.

## 4. Cross-Reactivity

The variability in the presentation and prevalence of “penicillin” allergy results from the ambiguity that is associated with the term. In fact, being labeled with a penicillin allergy cannot be linked with one prototypic presentation applicable to any agent from the beta-lactams class. In order to better characterize a penicillin allergy, it is important to understand the underlying pathophysiology or etiology that results in the presentation of the reaction. This will determine the inter-class cross-reactivity as well as the penicillins’ specific cross-reactivity [2].

**Figure 1:**
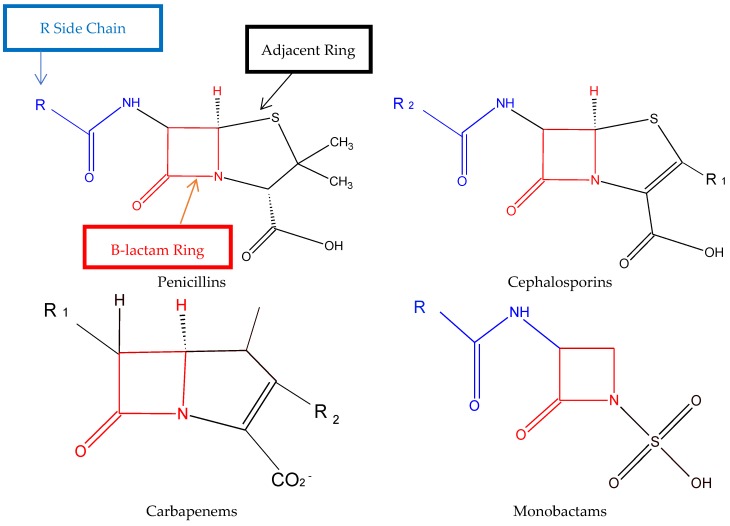
Structure and functional R-groups of different beta-lactam sub-classes.

As the name states, the structure of all beta-lactams consists at a minimum of a beta-lactam ring in addition to a potential adjacent or alpha ring and either 1 or 2 R side chains [25]. Penicillins contain an adjacent thiazolidine ring as well as 1 R side chain making each agent unique. Cephalosporins have an adjacent dihydrothiazine ring, but unlike penicillins, two side chains (R1 and R2) are within the structure. Carbapenems also contain 2 R side chains with a thiazodine ring slightly different than penicillin’s, while aztreonam, the monobactam, does not contain an additional ring but only 1 R side chain (Figure 1). The beta-lactam ring, additional rings and R side chains have all been reported to be potentially allergenic. The similarity between the R side chains as well as the degradation of the rings and the stability of the intermediate products determines the degree of cross-reactivity between beta-lactams [26,27].

In vivo, penicillin undergoes degradation under normal physiological conditions to produce reactive products that bind to self-proteins and thus illicit an immune response resulting in allergic reactions. The products, also termed antigenic determinants, are classified into major or minor ones relative to the quantity produced rather than their potential to elicit an immune response. The “major” determinant mostly consists of penicilloyl groups or conjugates, while several “minor” determinants consist of penicilloate, penicillanyl, penicillenate and others [3,27]. The aforementioned pathophysiology is the basis of cross reactivity between penicillins, the rationale behind skin testing and avoiding agents in the class if linked to the development of a severe anaphylactic IgE penicillin reaction. In contrast, the degradation process of cephalosporins results in more stable by-products that appear to be less immunogenic. This could explain the lower rates of cross-reactivity between different classes of beta-lactams as the physiological degradation process is altered but it also concurs with the potential immunogenic role of side chains [28,29].

Selective reactivity refers to a situation where a patient develops allergies to a specific agent within the penicillins class but can tolerate other agents within the same class. Selective reactivity is uncommon in the United States but more prevalent in Southern Europe such as Spain for unknown reasons [30]. The main agents that display selective reactivity within the penicillins class are the aminopenicillins such as amoxicillin and ampicillin. One study [31] included 54 patients with reported amoxicillin allergy and tolerance to penicillin based on skin testing or medication challenge. All of these patients were skin test negative to the major determinant penicilloyl and 96% were negative to a minor determinant benzylpenicillin. All 54 patients tolerated penicillin, making it the largest amoxicillin allergic penicillin tolerant study. It is for this reason that patients who report allergies to amoxicillin or ampicillin should also be tested for these agents in addition to the FDA approved penicillin skin testing (discussed in a different article in the same issue). The most probable reason for this selective reactivity is an immunogenicity induced by the R side chain specific to aminopenicillins.

Since the overall structure of penicillins is identical and major and minor determinants from the rings play a potentially significant role in immunogenicity, it is generally advised to avoid all penicillins in a severely penicillin allergic patient. It would also be prudent to pay particular attention to penicillins with similar side chains—typically agents within the same sub-class of penicillins, such as aminopenicillins, or the anti-staphylococcal or penicillinase-resistant penicillins, such as dicloxacillin, oxacillin and nafcillin. If a patient reports a particular allergy to an agent within a specific sub-class, caution should be taken prescribing another agent within the same sub-class due to similarities in side chains in addition to the core rings.

Numerous studies [32,33] have also demonstrated a cross-reactivity between penicillins and cephalosporins with similar side chains (discussed in a different article in the same issue). For example, ampicillin has a similar side chain compared to cefaclor, cefadroxil, cephalexin and cefradine. Amoxicillin shares similar side chains with the previously mentioned agents as well as cefprozil and cefatirizine. Penicillin shares a side chain closely related to cefoxitin. Many institutions have developed protocols that would group beta-lactams according to similar side chains, and recommendations to use or avoid agents in the presence of a certain beta-lactam allergy would be derived from these groups (Table 1). This concept is generally applied in patients with mild allergic reactions typically manifesting as rashes as opposed to life-threatening anaphylactic reactions. The latter group of patients was excluded in a large meta-analysis assessing the use of cephalosporins in penicillin allergic patients [34].

## 5. Unintended Consequences

The unintended consequences of antibiotic overuse and misuse in general are now widely recognized to impact individual patients, healthcare organizations and society as a whole [35,36,37]. Mislabeling patients with penicillin allergy can lead to the utilization of second line antibiotics that are costlier, broader spectrum and in many instances associated with multiple adverse events.

Penicillins are still considered first line therapy for many infectious diseases states as they are effective and relatively benign compared to other classes of antibiotics. Patients are thus expected to encounter more adverse effects and less optimal outcomes when using second line antibiotic classes. This in turn affects healthcare organizations as the length of stay increases in addition to the cost of care of these patients. The occurrence and treatment of multi-drug resistant organisms not only impacts care of individual patients and exhausts organizations’ resources but ultimately contribute to the spread of antimicrobial resistance in society as a whole.

Specifically, the impact of penicillin allergy documentation has now been assessed in multiple studies and is associated with increased prevalence of Methicillin-resistant *Staphylococcus aureus* (MRSA), Vancomycin-resistant enterococcus (VRE), and *Clostridioides difficile* (*C. diff*). A large retrospective matched case-control study [38] consisted of 51,582 cases with penicillin allergy, each with two controls matched for diagnosis category, gender, age, and admission date but without labeled penicillin allergy. The outcomes of interest of the study were the total hospital days and the prevalence of *C. diff*, MRSA and VRE between the two groups. On average, cases had 0.59 more hospital days compared to controls (95% CI 0.47–0.71), odds ratio for C. diff prevalence of 1.234 (95% CI 1.156–1.317), odds ratio for MRSA prevalence of 1.141 (95% CI 1.071–1.317) and odds ratio for VRE prevalence of 1.301 (95% CI 1.125–1.504). As expected, second line antibiotics, such as fluoroquinolones, clindamycin, and vancomycin, were more commonly utilized in cases. Similar results were reported in a retrospective chart review study [39] presented as an abstract at the American Academy of Allergy, Asthma and Immunology. Seven hundred and ninety-eight patients were evaluated that had VRE, MRSA or *C. diff*. Twenty-one percent of these patients had a documented beta-lactam allergy, which according to the authors was 1.75 times higher than the usual prevalence of this type of allergy for a similar patient population. Similarly, a large population based matched cohort study [40] in the United Kingdom found an association between penicillin allergy labeling and occurrence of MRSA and *C. diff*. Each case was matched to up to five controls based on age, gender and study entry time. A total of 64,141 cases were matched with 237,258 controls. The hazard ratio for developing MRSA in patients with penicillin allergy was 1.84 times the patients without allergy (95% CI 1.64–2.06). This finding remained consistent after adjusting for multiple variables such as age, gender, BMI, socio-economic status, smoking/alcohol use, and Charlson Comorbidity scores with a hazard ratio of 1.69 (95% CI 1.51–1.90). The hazard ratio for developing *C. diff* was 1.37 (95% CI 1.23–1.53) with adjusted hazard ratio of 1.26 (95% CI 1.12–1.40).

## 6. Conclusions

The undesired and drastic effects of antibiotic misuse is one of the current major public health threats. Mislabeling patients’ allergies, particularly penicillin allergy, is one of many ways antibiotics could be misused as it ultimately leads to the utilization of broader and alternative agents. It is important for clinicians to correctly characterize an allergy as well as be aware of cross-reactivity and appropriate management plans based on such allergy. This article focused on the basics of penicillin allergy and together with other topics within this issue will arm clinicians with the necessary guide to manage antimicrobials allergies appropriately.

## Figures and Tables

**Table 1:** Penicillins and other agents with similar R side chain.

Agent.	Agents with Similar Side Chains					
Penicillin	Cefoxitin	Cephalothin	Cefamandole			
Amoxicillin	Cefaclor	Cefadroxil	Cephalexin	Cefradine	Cefprozil	Cefatirizine
Ampicillin	Cefaclor	Cefadroxil	Cephalexin	Cefradine		
